# Kinematics and Kinetics of Taekwon-do Side Kick

**DOI:** 10.2478/v10078-011-0068-z

**Published:** 2011-12-25

**Authors:** Jacek Wąsik

**Affiliations:** 1 Institute of Physical Education, Jan Długosz University, Częstochowa, Poland

**Keywords:** taekwon-do, movement analysis, kick kinetics, power test

## Abstract

The aim of the paper is to present an analysis of the influence of selected kinematic factors on the side kick technique. This issue is especially important in the traditional version of taekwon-do, in which a single strike may reveal the winner. Six taekwon-do (International Taekwon-do Federation) athletes were asked to participate in this case study. Generally accepted criteria of sports technique biomechanical analysis were adhered to. The athletes executed a side kick three times (in Taekwon-do terminology referred to as yop chagi) in a way which they use the kick in board breaking. The obtained data were used to determine the mean velocity changes in the function of relative extension length of the kicking leg. The maximum knee and foot velocities in the Cartesian coordinate system were determined. The leg lifting time and the duration of kick execution as well as the maximum force which the standing foot exerted on the ground were also determined. On the basis of the obtained values, mean values and standard deviations were calculated. The correlation dependence (r=0.72) shows that greater knee velocity affects the velocity which the foot develops as well as the fact that the total time of kick execution depends on the velocity which the knee (r = −0.59) and the foot (r = − 0.86) develop in the leg lifting phase. The average maximum speed was obtained at the length of the leg equal to 82% of the maximum length of the fully extended leg. This length can be considered the optimum value for achieving the maximum dynamics of the kick.

## Introduction

The ability to break bricks or boards with bare hands or feet used to be associated with special, sometimes regarded as supernatural, abilities and predispositions of Eastern martial artists. The first attempts made at delivering a scientific biomechanical description of the techniques performed in martial arts go back to research projects conducted in the 1970s and 1980s (Vos and Binkhorse, 1966; Blum, 1977; Walker, 1975). Those studies described kinematic aspects of strikes and analyzed the process of breaking hard objects with bare hands. The stroboscopic method was used to register movement analyses which facilitated determining velocities, accelerations and strike execution times. The following years witnessed continuation of these studies (Wilk et al., 1982; Pieter and Pieter, 1985; Wąsik, 2007; 2009a), which attempted to describe the dynamic theory of strikes and a more detailed registration of kinematics of strikes. Research projects were also conducted with regard to protection gear worn by karate athletes (Smith and Hamill, 1986). Since Taekwon-do became an event in the summer Olympic Games an increasing number of studies on this art’s kicks and punches has been observed.

The front kick was analyzed in several aspects (Hwang, 1987), namely a) hip, knee and ankle muscle torque arrangement, b) sequence of actions of the dominant muscle group, c) muscle contraction type, and d) range of movement in regard to the efficient muscle torques applied. It was observed that in case of the high front kick thigh deceleration takes place as a result of actions dependent on the initial movement in the lower extremity and not the braking action as such (Sørensen et al., 1996). The roundhouse kick was also analyzed by several researchers. The mean roundhouse kick execution time was 0.35 and 0.30 seconds for male and female athletes of the Singapore top taekwon-do athletes (Boey and Xie, 2002). However, no connection between the shortest trajectory and peak velocity was found. The mean maximum velocity of this kick is between 12.84–16.26 m/s (Pieter and Pieter, 1995). Other researchers’ interests concerned the acceleration of the center of gravity, changes in the angles of body segments as well as changes in the momentum during execution of the roundhouse kick (Lee et al., 2001).

Description and analysis of a sports technique in relation to appropriate rules of biomechanics and with regard to its efficiency comprise the fundamentals of technical training which is directed at enhancing sport performance. This problem is of great importance in taekwondo, where a single strike might reveal the winner. In the Olympic Games, taekwon-do has been limited to sports combat whereas the traditional version of taekwon-do sports competition (International Taekwon-do Federation) comprises four competitive events, i.e. sparring, patterns, power tests and special techniques (Choi, 1983; 1995).

The power test involves breaking as many boards as possible by way of using a variety of strikes comprising two hand strike techniques and three kicking techniques, one of which is the side kick. The side kick (in taekwon-do terminology referred to as *yop chagi*) is a technique in which athletes tend to declare the highest number of broken boards. Thus, it is bound to affect the final score in each competition.

Hence, the aim of this study was to investigate side kick biomechanical optimization on the basis of kick execution time and the foot and knee velocity values obtained. Pursuant to the criteria of sports technique biomechanical analyses (Hay, 1993), and the measurement methods applied in taekwon-do research in particular (Wąsik, 2006; 2009b) four movement phases of the side kick have been specified in the present paper: starting posture (preparatory phase), shifting the back leg forward, lifting the leg and breaking (final phase). The following research questions arise:

1. At which moment is foot velocity the greatest ?
2. How does knee velocity affect foot velocity ?
3. How does the development of foot and knee velocities affect total time of the side kick?

Providing answers to these questions may result in developing a more efficient method of executing this particular kind of kick in taekwondo ITF sports competition power tests as well as in self-defense.

## Material and Methods

### Subjects

The study was based on 6 taekwon-do ITF (International Taekwon-do Federation) athletes comprising 1 female athlete and 5 male athletes. The researched group included European Junior Champions, Polish Junior Champions and other athletes who had practised taekwon-do for a minimum of 4 years. They train regularly 3 to 5 times a week.

### Protocol

For the purpose of the experiment, they were asked to adopt the same starting posture (in Taekwon-do terminology called Niunja So Palmok Degi Maki) and perform the side kick three times. The analysis covered 18 attempts altogether. The structure of the movement is presented in Pictures 1, 2 and 3. In this case study Smart-D system for complex movement analysis produced by BTS S.p.A. company was used. The system comprised six cameras emitting infrared rays, which in real time localized the markers fixed to the athlete’s body. These markers reflected the infrared rays emitted by the cameras. The system facilitated recording the picture of the movement of the competitor’s body and evaluation of the kinetic data obtained. The picture was recorded with accuracy of 0.3 – 0.45 mm and frequency of 120 Hz. Obtained data concerning the movement and speed of characteristic points on the athlete’s body were analyzed, which allowed to specify the indicators which define the structure of space and time of the athlete’s movement. In the analysis of particular segments of the technique the following factors were taken into consideration: v_z_ - speed of the foot with regard to Z axis, v_y_ - speed of the foot with regard to Y axis, v_kz_ - speed of the knee with regard to Z axis, v_ky_ - speed of the knee with regard to Y axis, t_kick_ – time from the moment of foot take off to the moment of full extension of the kicking leg, t_total_ – time from the moment of the movement of the athlete’s body to the moment of full extension of the kicking leg, F_r_ – the ground reaction force of the plant foot.

### Statistics

For the recorded parameters the average values and standard deviations (SD) were calculated. Correlation coefficients were determined between the foot and knee velocities and the kick duration time as well as the velocities of raising of the foot and knee. This correlation was verified at the significance level of p<0.05. All the statistical calculations were carried out with the use of MS Excel 2000.

## Results

### Starting posture

The athlete adopts the L-stance forearm guarding block (in Taekwon-do terminology referred to as *niunja sogi palmok debi maki*) with the right foot moved forward. According to taekwondo rules (Choi, 1995) in this stance 70% of the body weight should rest on the back foot and 30% on the front one. Both feet should be slightly pointed inwards and the toes of the foot at the front should be lined up with the heel of the back foot. Both knees are slightly bent. The term ‘starting posture’ comprises information on the stance and the place where the attempted attack starts.

### Shifting the back leg forward

The athlete moves the back foot forward in the direction of the intended impact. This results in a slight rise of the COG. The hands are held up in a guard. When the feet have touched the ground, the ankle joint tenses and the athlete energetically pushes the right foot off the ground.

### Lifting the leg

As a result of the right foot take-off the force pushes the foot upwards. Further movement is facilitated by the muscles of the lower limb taking control over the movement. Thus, the knee and hip joints are extended. This phase witnesses the greatest velocity of the foot traveling upwards with the mean velocity being v_y_= 5.10 m/s. On average it is in the 82% of the full leg extension when the foot reaches its peak velocity, whose mean is v_z_= 5.65 m/s (Picture 4). The average time of lifting the leg until the moment of its full extension is 0.39 s.

#### Final phase

The kicking foot is extended in the ankle joint. The athlete has had to balance his/her body in such a way so as to make sure that the foot planted on the ground has remained the only point of his/her body being in contact with the ground. Total time of kick execution (from the starting posture to the final phase) produced an average time of t=0.71 s.

The average values of the kinetic parameters are presented in Table 2.

## Discussion

It is indeed a difficult task to compare the obtained results with the data in other research projects as different methods are used to register speed and force of the kick. However, comparing the results obtained in different research projects can still provide some feedback on certain general trends.

The velocity of the side kick which Wilk et al. (1982) obtained when studying karate athletes (no particular karate style was specified) was between 9.9 and 14.4 m/s. In the research involving top American taekwon-do athletes Pieter and Pieter (1995) obtained the velocity of the side kick of 5.20–6.87 m/s. The mean maximum velocity of the side kick determined in that research was 5.65 m/s. Such results show a certain difference in velocities obtained by karate and taekwon-do athletes. It can be assumed that such differences can be attributed to different ways of execution of the kick in those martial arts.

Picture 5 shows the correlation between maximum foot velocity and maximum knee velocity during execution of the side kick (r=0.72). This dependence shows that higher knee velocity increases the velocity developed by the foot. This means that while working on improvement of the side kick technique aiming at increasing foot velocity attention needs to be paid to knee velocity which needs to correspond to the movement of the foot. Picture 6 presents the correlation between the duration of the side kick and the maximum velocities of the knee v_ky_ (r=−0.59) and the foot v_y_ (r=−0.86) in relation to axis Y. This implies that a short duration of the side kick will depend on the maximum velocity achieved by the knee and foot in the leg lifting phase. The total duration of the side kick comprises the time between the starting posture and shifting the back leg forward as well as the leg lifting time. These factors are presented in Picture 7.

The conducted measurement of the absolute force of the side kick showed that taekwon-do athletes obtained values ranging from 390 to 461 N (Pieter and Pieter, 1995). However, there was no information provided on the distance from the aim of the impact which they covered while executing the kicks. That research also shows that there is a strong dependence between the force of the side kick and the weight of an athlete. Not only does a greater weight add to a greater force, but it also results in an increase of the kinetic energy (Pieter et al., 1987). Knowledge of these facts proves considerable importance in board breaking as in this particular case the time and a high level of average energy production are not as important as the maximum energy at the very moment of the impact (Stull and Barham, 1990).

Well adjusted distance affects the force of the kick which can be obtained (Falco et al., 2009). The mean maximum velocity calculated in the research was achieved at the length of the leg equal to 82 % of the maximum leg extension. This length can be regarded as the optimum value in this kicking technique making it possible to achieve maximum dynamics of the kick. Thus, using the kinetics of the kick in full is only possible in cases when the aim of the impact is located at the optimum distance given for this particular kind of kick. According to the equation $F=\frac{m\cdot v^{2}}{2s}$ it can be assessed that at the moment when the velocity of the foot reaches the maximum value, the force of the impact will also be the greatest (Hay, 1993; Wąsik, 2007). The force of the kick assessed as presented above with the mean maximum velocity and mean athlete’s body weight (provided the total athlete’s body weight is engaged in the movement) would amount to F ≈ 1020 N. In case of such assessment a mistake in the distance which equals to 10 % would mean a decrease in the power of the kick down to F ≈ 512 N.

Thus, a precise assessment of the distance along with the correct time of the moment of impact constitute a very important aspect of information for taekwon-do athletes, especially those taking part in power breaking events, where the aim of the impact is a fixed board.

In conclusion, board breaking requires great maximum velocity at the moment of impact, which is usually achieved at the cost of attack duration (Stull and Barham, 1990). However, in cases in which the aim is to obtain points in sports competition, athletes will need to focus on reducing the duration of the kick and increasing mean kick velocity.

In pursuit of perfection, further research will most certainly be required in order to determine duration of the side kick, influence of reaction time on the achieved power of the kick as well as analyses of taekwon-do kicks during real-time fighting, which will make it possible to specify which variables of this particular kick affect its efficiency. The present study is an introduction to further more detailed research into this particular issue. The results and discussion presented herein can be regarded as comparative material for other researchers.

## Figures and Tables

**Picture 1:**
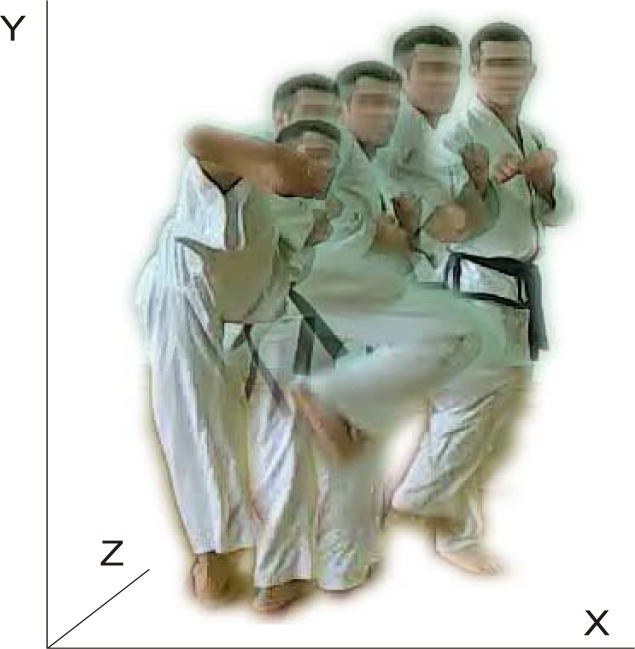
Diagram of the side kick movement structure (in taekwon-do terminology referred to as yop chagi) – front view

**Picture 2:**
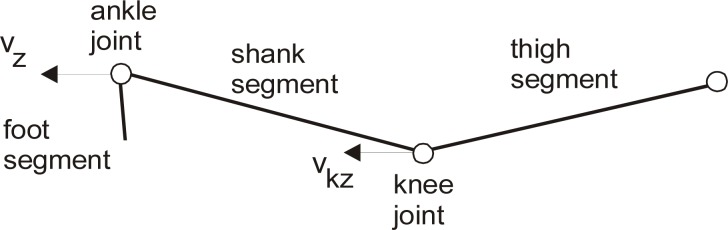
General view presenting relevant joints and segments of the kicking leg.

**Picture 3:**
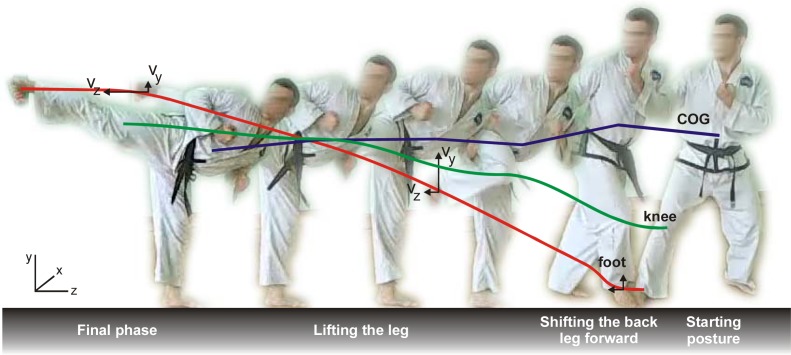
Yop chagi movement structure diagram – side view. Red line – trajectory of the foot; green line – trajectory of the knee; blue line – transition of the Centre of Gravity (COG).

**Picture 4:**
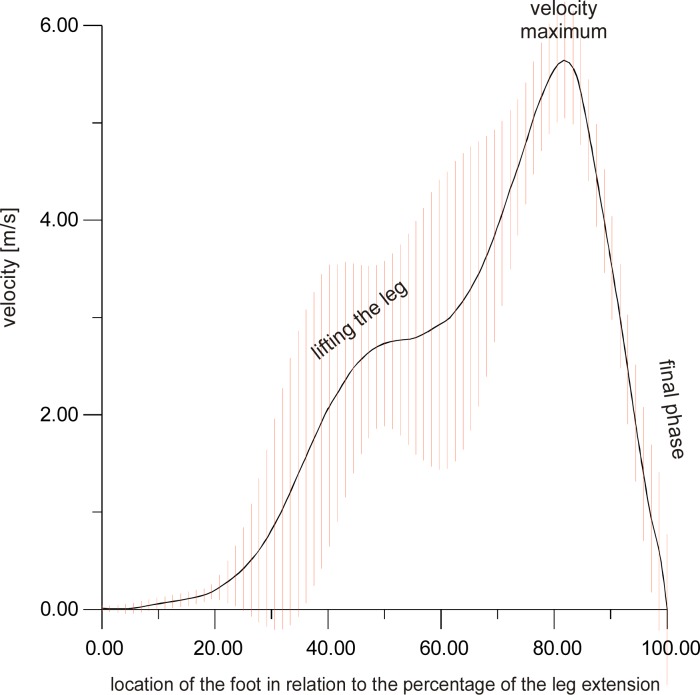
Average change of foot velocity depending on leg flexion while executing yop chagi in realtion to axis Z.

**Picture 5:**
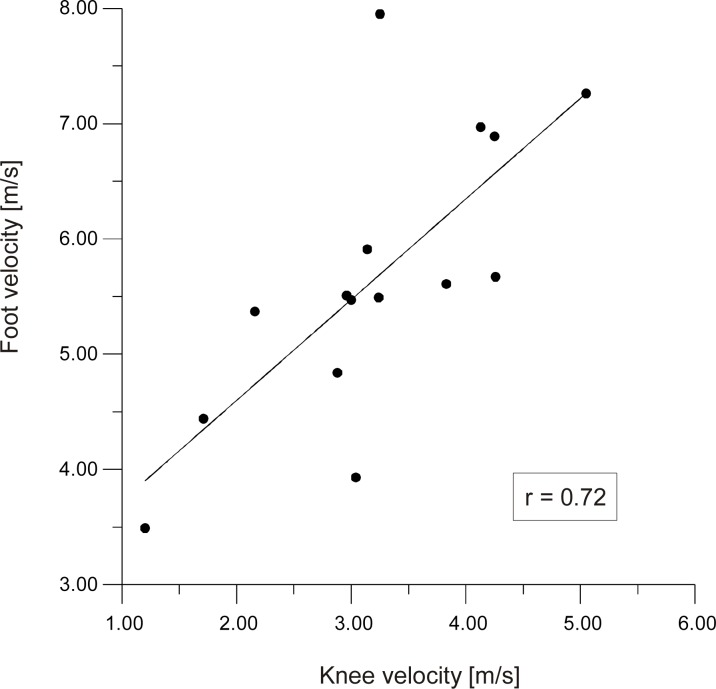
Correlation between maximum velocity of the foot and knee during the side kick

**Picture 6:**
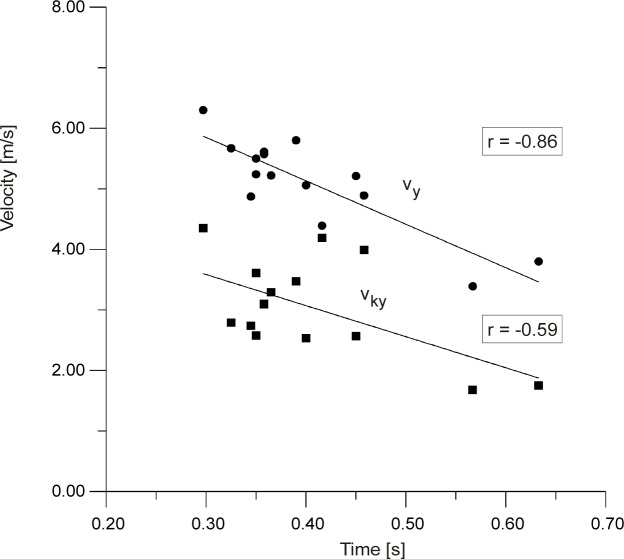
Correlation between the velocity of the traveling foot (v_y_), knee (v_ky_) and kick execution time (t_total_)

**Picture 7:**
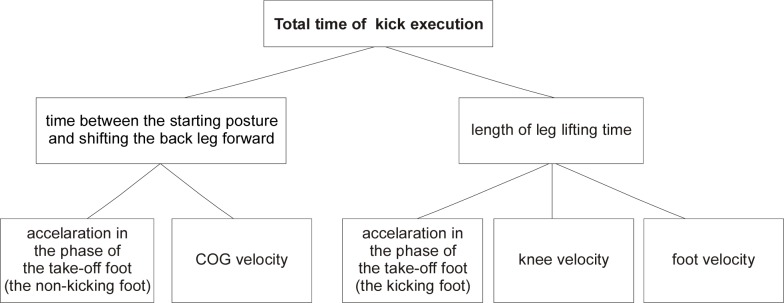
Factors affecting the side kick execution time

**Table 1:** Physical characteristics (means ± SD) of male and female taekwondo athletes.

Variables	Athletes (n=6)	SD	Range
Age (years)	16.50	0.71	15 – 18
Weight (kg)	64.14	7.04	50 – 80
Height (cm)	176.50	4.64	162 – 182

**Table 2:** Biomechanical parameters influencing the efficiency of the kick

Variables	Average	SD	Range
velocity of the foot with regard to Z axis v_z_ [m/s]	5.65	1.22	3.49 ÷ 7.95
velocity of the foot with regard to Y axis v_y_ [m/s]	5.10	0.76	3.39 ÷ 6.30
velocity of the knee with regard to Z axis v_kz_ [m/s]	3.21	1.02	1.2 ÷ 5.05
velocity of the knee with regard to Y axis v_ky_ [m/s]	3.04	0.79	1.68 ÷ 4.35
time from the moment of the foot takeoff to the moment of the full extension of the kicking leg t_kick_ [s]	0.39	0.09	0.29 ÷ 0.63
time from the moment of the movement of the athlete’s whole body to the moment of the full extension of the kicking leg t_total_[s]	0.71	0.11	0.46 ÷ 0.89
the ground reaction force of the plant foot F_r_[N]	1253	248	1003 ÷ 1779
